# Novel Antibody Exerts Antitumor Effect through Downregulation of CD147 and Activation of Multiple Stress Signals

**DOI:** 10.1155/2022/3552793

**Published:** 2022-11-04

**Authors:** Keisuke Fukuchi, Kayoko Nanai, Hiroshi Yuita, Chikako Maru, Jun Tsukada, Masato Ishigami, Yoko Nagai, Yoko Nakano, Chigusa Yoshimura, Kozo Yoneda, Masato Amano, Kensuke Nakamura, Yoko Oda, Haruyuki Nishigohri, Shoji Yamamoto, Yusuke Ohnishi-Totoki, Koichiro Inaki, Hironobu Komori, Rika Nakano, Yoshiyuki Kanari, Atsuko Nishida, Yumi Matsui, Satoko Funo, Sayako Takahashi, Toshiaki Ohtsuka, Toshinori Agatsuma

**Affiliations:** ^1^Oncology Research Laboratories I, Tokyo, Japan; ^2^Clinical Development Department, Daiichi Sankyo Co., Ltd, Tokyo, Japan; ^3^Biologics & Immuno-Oncology Laboratories, Tokyo, Japan; ^4^Drug Metabolism & Pharmacokinetics Research Laboratories, Tsukuba-shi, Japan; ^5^New Modality Research Laboratories, Yokohama, Japan; ^6^Research Function, Tokyo, Japan; ^7^Oncology Research Laboratories II, Daiichi Sankyo Co., Ltd, Tokyo, Japan; ^8^Translational Research Department, Tokyo, Japan; ^9^Biological Research Department, Daiichi Sankyo RD Novare Co., Ltd, Tokyo, Japan

## Abstract

CD147 is an immunoglobulin-like receptor that is highly expressed in various cancers and involved in the growth, metastasis, and activation of inflammatory pathways via interactions with various functional molecules, such as integrins, CD44, and monocarboxylate transporters. Through screening of CD147-targeting antibodies with antitumor efficacy, we discovered a novel rat monoclonal antibody ^#^147D. This humanized IgG4-formatted antibody, h4^#^147D, showed potent antitumor efficacy in xenograft mouse models harboring the human PDAC cell line MIA PaCa-2, HCC cell line Hep G2, and CML cell line KU812, which featured low sensitivity to the corresponding standard-of-care drugs (gemcitabine, sorafenib, and imatinib, respectively). An analysis of tumor cells derived from MIA PaCa-2 xenograft mice treated with h4^#^147D revealed that cell surface expression of CD147 and its binding partners, including CD44 and integrin *α*3*β*1/*α*6*β*1, was significantly reduced by h4^#^147D. Inhibition of focal adhesion kinase (FAK), activation of multiple stress responsible signal proteins such as c-JunN-terminal kinase (JNK) and mitogen-activated protein kinase p38 (p38MAPK), and expression of SMAD4, as well as activation of caspase-3 were obviously observed in the tumor cells, suggesting that h4^#^147D induced tumor shrinkage by inducing multiple stress responsible signals. These results suggest that the anti-CD147 antibody h4^#^147D offers promise as a new antibody drug candidate.

## 1. Introduction

*Cancer* is a leading cause of death worldwide and remains difficult to cure, although various therapies have been developed over recent decades [1]. In particular, pancreatic ductal adenocarcinoma (PDAC) and hepatocellular carcinoma (HCC) are rarely treated curatively, with a short survival period from diagnosis, especially once those are advanced/metastatic [2]. Even though recent advances of immune-checkpoint inhibitors (ICIs) could offer better survival outcomes than pre-existing standard-of-care in several other cancer types, these inhibitors have provided limited clinical success for HCC and PDAC by monotherapy, while the combination of ICIs with other drugs has been actively tested in clinics [3, 4]. So far, ICI-combination therapy has no significant success in unresectable PDAC, which is potentially due to its disease biology such as immunosuppressive tumor microenvironment [5]; therefore, a combination of cytotoxic chemotherapy has remained as a mainstream in its practice [6]. For unresectable HCC, atezolizumab plus bevacizumab combination therapy was approved as first-line therapy based on the significant improvement in overall survival as compared with sorafenib [7]. Although antiangiogenics, including multiple kinase inhibitors, have been the key drugs for metastatic HCC, the response rate is less than 20%. In addition, there are few treatment options for HCC patients with poor hepatic reservoirs, indicating that new treatment options with better safety and efficacy profiles would be needed for HCC patients [8].

Several cancers are known to acquire sustained cell growth and other malignant phenotypes by gaining a single mutation or genomic alteration in certain oncogenes, which are known as driver mutations. Such mutations have been identified in limited populations of blood and lung cancers, and the stepwise accumulations of multiple mutations in oncogenes and/or tumor suppressor genes are the mainstay in cancer development, which can transform normal cells into malignant cells by stepwise acquisition of malignant phenotypes and finally lead to intractable disease [9]. In chronic myeloid leukemia (CML) with a *BCR-ABL* fusion gene, treatment with tyrosine kinase inhibitors (TKis) specifically targeting BCR-ABL results in remission and prolongation of survival in most patients; however, 60–70% of CML patients may become insensitive to the drug by developing acquired resistance in the chronic phase [10]. In such resistant cases, a downstream pathway and a parallel pathway bypassing the growth inhibitory signal including the basigin signaling pathway are activated [10, 11]. Conventional molecular therapies targeting a single oncoprotein have therefore failed to deliver longitudinal efficacy against the cancer types with multiple gene mutations/alterations leading to the diverse activation of cancer-related signaling pathways [12–14].

CD147, also known as basigin or ECM metalloproteinase inducer, is a plasma membrane-expressed glycoprotein belonging to the immunoglobulin superfamily. This glycoprotein is expressed in a variety of cells and is highly expressed in many types of cancer. High expression of CD147 is reportedly associated with a poor prognosis in several cancer types, including PDAC and HCC [15–17]. No enzymatic domain is present in the intracellular part of CD147. On the other hand, CD147 contains two Ig-like domains (Ig-LDs), which can interact with various proteins including CD44, integrins, and monocarboxylate transporter 1 (MCT1). CD44 and integrins are known to bind to their ligands, ECM proteins, to promote cell survival, proliferation, and metastasis. Increased expression of these molecules correlates with a poor prognosis in several cancer types [18–20]. CD44 and integrins are internalized and degraded in lysosomes once those are activated by interactions with their ligands, while coexpressed CD147 attenuates the degradation of CD44 and integrins and supports recycling on the cell surface by binding to these molecules. In the recycling process via CD147, the intercellular C-terminal domain of CD147 that could interact with Hook1 is involved in protein trafficking to the cellular membrane and those recycling [21]. Overall, CD147 acts as a protein chaperone in these processes through supporting stabilization and recycling [22].

MCT1 is a membrane protein involved in excreting lactate generated by glucose metabolism to maintain an appropriate intercellular pH [23]. Cell surface expression of MCT1 is reported to depend on CD147 expression, which stabilizes MCT1 and facilitates its membrane translocation [24, 25]. CD44, integrins, and MCT1 have also been reported to play key roles in cell adhesion, survival, and metabolism in proliferating cancer cells, and those expressions correlate with a poor prognosis in cancer. These proteins are therefore considered as candidate target molecules for cancer therapy [26–29].

With this background, we identified a new CD147 antibody that affects tumorigenic signals transduced via interactions between CD147 and other proteins such as CD44, integrins, and MCT1 to exhibit its antitumor activity in this study. Here, we also report the therapeutic potential of the anti-CD147 antibody *in vivo* and the mechanistic analyses to elucidate the effects of this antibody on binding molecules as well as related signals for cell growth and death.

## 2. Materials and Methods

### 2.1. Ethical Statement

All animal studies were conducted with the approval and in accordance with the guidelines of the Institutional Animal Care and Use Committee of Daiichi Sankyo Co., Ltd. (Tokyo, Japan).

### 2.2. Cells

Human cancer cell lines (PANC-1, PDAC cell line; MIA PaCa-2, PDAC cell line; BxPC-3, PDAC cell line; Hep G2, HCC cell line; and KU812, CML cell line) and the SP2/0-ag14 murine myeloma cell line were purchased from ATCC (Manassas, VA, US).

### 2.3. Antibody Generation and Screening

Hybridomas producing the monoclonal antibody were generated from mice or rats after immunization with recombinant human CD147 protein (Creative BioMart, Shirley, NY, US) or CD147-expressing cells by the myeloma fusion method. Antibodies showing binding activity to recombinant CD147 protein were screened by the ELISA. The screened anti-CD147 antibodies were purified from the hybridoma supernatant and tested for antitumor effects on MIA PaCa-2 and PANC-1 PDAC cell line xenograft models. The detailed antibody generation and screening process were described in the supporting information. For use in humans, a rat anti-CD147 antibody clone ^#^147D showing *in vivo* efficacy was humanized by CDR (complementarity-determining region) grafting [30, 31] to minimize immunogenicity, and a humanized IgG4-formatted antibody clone with heavy chain mutation S228P (IgG4Pro) [32] for hinge stabilization was obtained and named h4^#^147D.

### 2.4. Surface Plasmon Resonance (SPR)

The binding affinity of h4^#^147D against recombinant CD147 proteins was analyzed by SPR using a Biacore T200 system (Cytiva, Tokyo, Japan). Purified h4^#^147D was immobilized to a sensor chip using a human antibody capture kit (GE Healthcare, Chicago, IL, US), and then, each recombinant human or monkey CD147 protein was injected. Dissociation constant (*K*_D_) values were calculated in 1 : 1 fitting models using Biacore T200 Evaluation software (Cytiva).

### 2.5. Xenograft Study

Five-week-old female NOD SCID mice (*n* = 5 or 6 per group) were injected subcutaneously in the dorsal right flank with 5 × 10^6^ of MIA PaCa-2, Hep G2, or KU-812 cells in a 50% Matrigel solution (PBS/Matrigel, 1 : 1: v/v; Corning, NY, US). When the tumor had grown to a mean volume ≥100 mm^3^, mice were randomized into treatment and control groups based on tumor volumes and injected intravenously or intraperitoneally with the anti-CD147 antibody either once or weekly. Estimated tumor volumes were calculated according to the following formula:

Estimated tumor volume (mm^3^) = 0.5 × tumor length (mm) × (tumor width (mm))^2^.

Tumor growth inhibition (TGI) was calculated as a percentage according to the following formula: 100 (1 × (average tumor volume of the treatment group)/(average tumor volume of the control group)), and tumor volumes were compared between control and treatment groups.

### 2.6. Crystal Structure Determination

The CD147 extracellular domain (residues 22–205) with an N-terminalHis-tag and tobacco etch virus protease site was expressed in *Escherichia coli* and purified by Ni-affinity chromatography and subsequent gel-filtration chromatography using a HiLoad 16/600 Superdex 75 pg column (Cytiva), followed by tag cleavage, further Ni-affinity chromatography, and buffer exchange. The Fab' fragment of h4^#^147D was prepared from IgG produced in 293 F cells by digestion with pepsin and subsequent alkylation with iodoacetamide under reducing conditions. The complex of the CD147 extracellular domain and h4^#^147D Fab' (CD147-Fab' complex) was prepared by mixing and purified by gel-filtration chromatography using Superdex 10/300 GL Increase columns (Cytiva). Those fractions containing the CD147-Fab' complex were collected and concentrated up to 13 g/L in Tris/HCl buffer (50 mM Tris/HCl (pH 7.5), 50 mM NaCl). The CD147-Fab' complex was crystallized by the vapor diffusion method with a 1 : 1 mixture of protein solution and reservoir solution (0.1 M sodium malonate, pH 7.0, 12% (w/v) PEG 3350). Crystals obtained after 1 week were immersed in a reservoir solution supplemented with PEG 400 before freezing in liquid nitrogen. X-ray diffraction data were collected at the beamline PF-BL17 A of the Photon Factory synchrotron radiation facility (Tsukuba, Ibaraki) in KEK (High Energy Accelerator Research Organization, Tsukuba, Japan), and diffraction intensities were processed using XDS [33] and MOSFLM [34]. Molecular replacement was performed using PHASER [35] and the reported structure of CD147 (PDB ID : 3B5H) and a homology model of the Fab' fragment as search models. The obtained structural model of the CD147-Fab' complex was refined to Rwork = 0.24 and Rfree = 0.28 at a resolution of 2.3 Å using REFMAC5 [36] and Coot [37]. Coordinate and structural factors were deposited in the Protein Data Bank (accession code: 7XY8).

### 2.7. Flow Cytometry

Cells were prepared using a tumor dissociation kit (Miltenyi Biotec, Rheinisch-Bergischer Kreis, Germany) from xenograft tumor tissues at 24 or 72 h after administration of the anti-CD147 antibody. Fluorescent signals were detected using a CantoII flow cytometry analyzer (BD Biosciences, Franklin Lakes, NJ, US) for cells stained with detection antibodies (Table 1). To remove dead cells, 7-AAD staining was performed, and 7-AAD-positive cells were removed from analysis. To analyze human cancer cells in xenograft tumors, antihuman CD9-positive cells were analyzed. Expression levels of each molecule were calculated as percentages of nontreated samples after subtraction of isotype control sample signals as background signals.

### 2.8. Western Blot Analysis

Tumor lysates were prepared using a GentleMACS system (Miltenyi Biotec) from MIA PaCa-2 xenograft tumor tissues at 24 and 72 h after administration of the anti-CD147 antibody. Lysates were analyzed using a protein capillary electrophoresis device, WES or Peggy Sue (ProteinSimple San Jose, CA, US). Molecules to be analyzed and antibodies used for detection are shown in Table 2. After electrophoresis, luminescent signals estimated from the molecular weight of the target molecule were standardized with the *β*-actin protein expression signal. Analysis was performed for h4^#^147D-treated groups in comparison with isotype control antibody-treated (after 24 h) or nontreated (after 72 h) groups.

### 2.9. Generation of KLF5-Expressing Cells

To generate a KLF5 expression retrovirus vector, a DNA fragment containing the genetic sequence of KLF5 (Refseq ID : NM_001730.4; Genscript clone ID : OHu21278 C) was inserted into a cloning site of a retroviral vector (Genscript Biotech, Piscataway, NJ, US). Retroviruses produced by retrovirus vector-transfected GP2-293 cells (Takara Bio, Shiga, Japan) were infected into MIA PaCa-2 cells, and then, those cells in which retroviruses had integrated into the chromosome were selected by drug selection. MIA PaCa-2 cells produced in the same procedure using retroviral vectors without KLF5 were used as mock cells and KLF5-negative cells.

The generated retrovirus-infected cells were analyzed by western blotting to confirm the expression of KLF5 protein and were used for later experiments. A capillary protein electrophoresis device (Wes; ProteinSimple) was used for western blotting analysis. Anti-KLF5 antibodies (AF3758; R&D Systems, Minneapolis, MN, US) were used to detect KLF5 protein. The anti-*β*-actin antibody (^#^4967L; Cell Signaling Technology, Danvers, MA, US) was used to detect *β*-actin protein as an endogenous control. Images of luminescent signals in protein electrophoresis were obtained using Compass software (ProteinSimple).

### 2.10. Pharmacological Evaluation in Mice Bearing KLF5-Positive MIA PaCa-2

KLF5 expression and mock lines of MIA PaCa-2 were evaluated for antitumor effects of h4^#^147D in mouse xenograft models, as well as parental MIA PaCa-2 cells. Each cell was grouped 3 days after implantation subcutaneously in mice, and h4^#^147D was administered at 1 mg/kg via the tail vein once weekly for a total of two doses. Antitumor effects were evaluated using the h4^#^147D nontreated group as the control group.

### 2.11. Statistical Analysis

For observed change in the tumor volume and protein expression level in the tumor with each compound or antibody treatment, a parametric Dunnett test was conducted between the treatment (*T*) and control (C) groups to evaluate the efficacy of the antibody. A 2-sided*P* value of <0.05 was considered statistically significant. Hypothesis testing of the Spearman rank correlation coefficient (the null hypothesis correlation coefficient is 0) was conducted among the *T* and C groups to evaluate the expression level of SMAD4 protein-dependent antitumor activity of antibodies. All statistical analyses were performed using REDPOST/BI (SAS System Release 9.2; SAS Institute Inc, Cary, North Carolina, USA).

## 3. Results

### 3.1. Binding Profile of the Obtained Anti-CD147 Antibody, h4^#^147D Antibody

Among the five humanized anti-CD147 antibodies, the h4^#^147D-humanizedIgG4-formatted antibody showed the best monkey CD147 cross-reactivity and the potent anti-tumor effect was obtained from the rat anti-CD147 antibody clone ^#^147D, as described in Materials and Methods. The detailed process of antibody generation and screening was described in supporting information as “antibody generation and screening.” The features of h4^#^147D, including binding affinity to human and cynomolgus monkey CD147, are summarized in Table 3. Due to the use of the IgG4 format with lower affinity to Fc*γ* receptors and C1q than the IgG1 format [32], no significant effector functions such as antibody-dependent cellular cytotoxicity (ADCC), complement-dependent cell toxicity (CDC), or antibody-dependentcell-mediated phagocytosis (ADCP) were observed in h4^#^147D (Table 3, Supplemental Figures S3–S5). As CD147 is expressed in normal tissues, aiming to decrease the risk of adverse events in CD147 expressed normal tissues by figure S$the antibody mediated effector functions, the IgG4 format was selected for the final format of the anti-CD147 antibody Kd values for the binding of h4^#^147D to human and cynomolgus monkey orthologues were similar in the order of 10^−9^ M. X-ray crystal structure analysis revealed that this binding occurred at a site in membrane-proximalIg-LD near the junction with the adjacent IgC2 domain (Supplemental figure S2).

### 3.2. Pharmacological Activity of the Anti-CD147 Antibody

**Figure 1:**
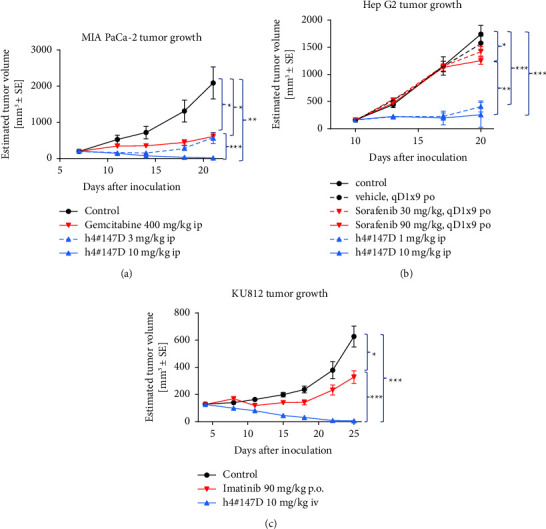
Antitumor efficacy of h4^#^147D compared to the standard anticancer drugs gemcitabine, imatinib, and sorafenib in xenograft NOD SCID mice harboring MIA PaCa-2 PDAC (a), Hep G2 HCC (b), and KU812 CML cells (c), respectively. Injection of h4^#^147D was performed once, on the day of group allocation. Tumor size was measured twice weekly using calipers. Data represent the mean ± SEM. *n* = 5 or 6 mice per group. Asterisks indicate significant differences between each group connected by a line (^*∗∗∗*^*P* < 0.001, ^*∗∗*^*P* < 0.01, and ^*∗*^*P* < 0.05).

Antitumor effects of h4^#^147D were evaluated in xenograft mouse models of PDAC and HCC and CML and were compared with those of the respective standard-of-care (SOC) drugs (Figure 1(a)).

MIA PaCa-2-grafted mice were used as a PDAC model, with gemcitabine as the SOC drug for comparison. In MIA PaCa-2 xenograft models, gemcitabine showed a partial anti-tumor effect with 71% TGI and no complete tumor regression in any of the five tested mice at a dose of 400 mg/kg, whereas h4^#^147D showed potent antitumor effects with tumor regression as 99% TGI at a dose of 10 mg/kg. Complete tumor regression was observed in four of the five mice in the h4^#^147D-treated group.

Hep G2-grafted mice were used as an HCC model. The antitumor efficacy of h4^#^147D was compared with that of the SOC drug for HCC therapy, sorafenib (Figure 1(b)). Partial inhibition of tumor growth was observed in xenograft models of Hep G2 in the sorafenib-treated group with 8% TGI at 30 mg/kg and 28% at 90 mg/kg. On the other hand, potent antitumor efficacy with 76% TGI was observed in the group treated with h4^#^147D at 1 mg/kg, and 85% TGI was observed in the group treated with 10 mg/kg. Complete tumor regression was observed in two of the five mice in the h4^#^147D-treated group.

KU812-grafted mice were used as the CML model, with imatinib as the SOC drug. In the xenograft mouse model with KU812 (Figure 1(c)), TGI was 48% in the imatinib-treated group and 99% in the h4^#^147D-treated group. The imatinib-treated group showed no instances of complete tumor regression, whereas complete tumor regression was observed in four of the five mice in the h4^#^147D-treated group.

Given these results, the h4^#^147D anti-CD147 antibody appears to exhibit potent anti-tumor efficacy against intractable cancers with low sensitivity to SOC drugs.

### 3.3. Effect of h4^#^147D on the Molecular Chaperone Function of CD147

**Figure 2:**
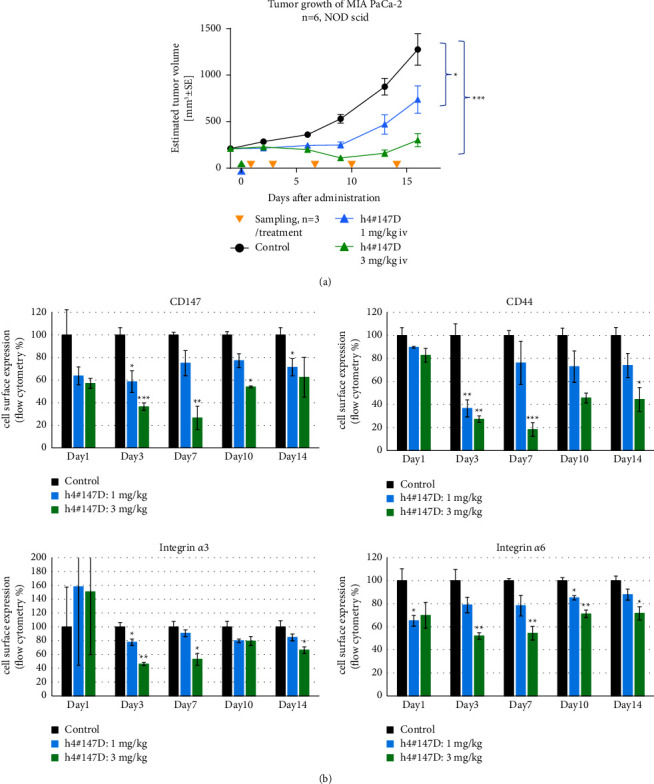
Cell surface expression of CD147 and its binding partners on MIA PaCa-2cell-derived xenograft tumors after administration of h4^#^147D. (a) Antitumor effect of h4^#^147D in the mouse xenograft model. Five-week-old NOD SCID female mice were subcutaneously injected with MIA PaCa-2. After tumor volume reached 200 mm^3^, animals were randomized into groups of 6 mice and treated with either 1 or 3 mg/kg body weight h4^#^147D (i.v., single injection at day 0; 1 mg/kg blue arrow; 3 mg/kg green arrow). Vehicle-treated mice were used as a control group. Data show the mean and SEM of the estimated tumor volume. Asterisks indicate significant differences between each group connected by a line (^*∗∗∗*^*P* < 0.001 and ^*∗*^*P* < 0.05). (b) Protein expression analysis using flow cytometry. After treatment with h4^#^147D, expressions of CD147, CD44, integrin *α*3, and integrin *α*6 were detected using the antibodies listed in Table 1 for cells prepared from tumors (*n* = 3) on days 1, 3, 7, 10, and 14. Measured signals are shown as the percentage of the average signal from the control group. Graphs represent the mean per group (*n* = 3 mice per group per time point), and error bars represent SEM. Asterisks indicate significant differences between the control group and antibody-treated groups (^*∗∗∗*^*P* < 0.001, ^*∗∗*^*P* < 0.01, and ^*∗*^*P* < 0.05).

CD147 is reported to act as a molecular chaperone helping to recycle several membrane proteins, including CD44 and integrins [21, 38, 39]. To elucidate the effects of h4^#^147D on the molecular chaperone function of CD147, expressions of CD147 and its binding proteins on the cell surface were analyzed using flow cytometry for human cancer cells obtained from tumor samples of xenograft mice bearing MIA PaCa-2 after h4^#^147D treatment. In vivo tumor growth was inhibited in a dose-dependent manner by h4^#^147D treatment, suggesting that h4^#^147D exerts antitumor activity in this model (Figure 2). At 16 days after administration of h4^#^147D, 42% and 76% TGIs were observed in the 1 mg/kg h4^#^147D-treated group and 3 mg/kg h4^#^147D-treated group, respectively. Reduction of CD147 and its binding membrane proteins, including CD44, integrin *α*3, and integrin *α*6, were notable on the surface of MIA PaCa-2 cells during 14 days after administration of the antibody, and modulations were seen in a dose-dependent manner (Figures 2(a)–2(d). This suggests that h4^#^147D inhibits the molecular chaperone function of CD147.

### 3.4. Effects of h4^#^147D on Molecules Related to Cell Growth and Survival Signals

**Figure 3:**
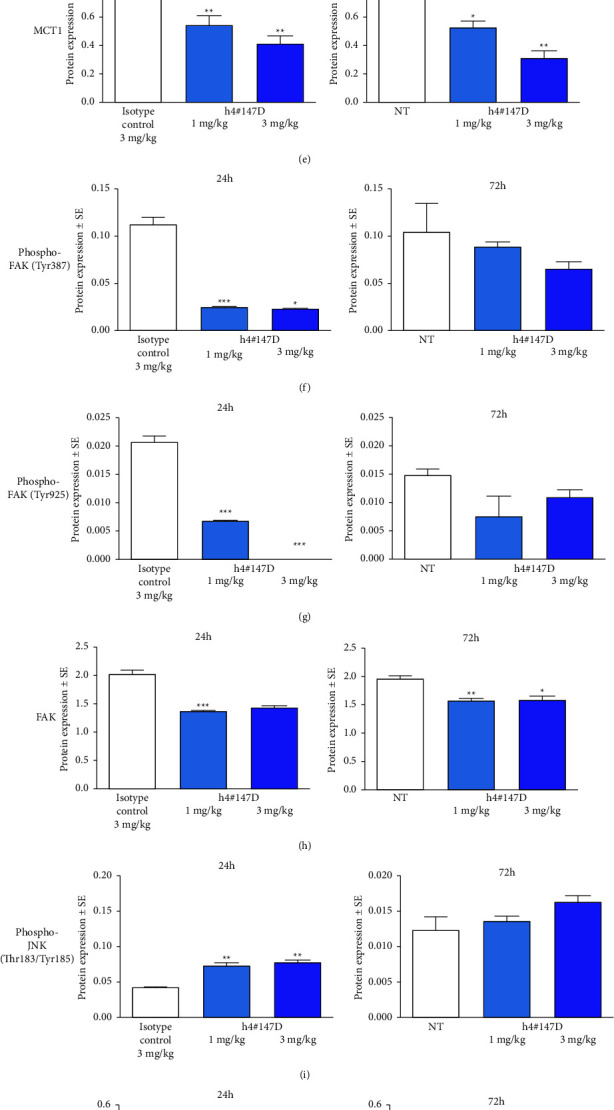
Protein expression of CD147, its binding partners, and downstream molecules in MIA PaCa-2 tumors after administration of the h4^#^147D antibody. Bar graphs show the mean ± SEM, *n* = 3 in all cases for the expression and luminescence signal ratio of CD147 (a), CD44 (b), integrin *α*3 (c), integrin *α*6 (d), MCT1 (e), phosphorylated-FAK, (Tyr397 (f) and Tyr925 (g)), total FAK (h), phosphorylated JNK (i), phosphorylated c-Jun (j), phosphorylated HSP27 (k), PAI-1 (l), and cleaved caspase-3 (m) to *β*-actin in tumor lysate samples from control tumors (isotype control-treated at 24 h or nontreated at 72 h and h4^#^147D-treated tumors (1 mg/kg or 3 mg/ml). Asterisks indicate significant differences between the isotype control-treated group at 24 h or nontreatment group (NT) as a control at 72 h and antibody-treated groups (^*∗∗∗*^*P* < 0.001, ^*∗∗*^*P* < 0.01, and ^*∗*^*P* < 0.05).

The effects of h4^#^147D administration on protein expression of CD147, its binding partners, CD44, integrin *α*3, integrin *α*6, and MCT1, and downstream signaling molecules were analyzed using tumor samples derived from the MIA PaCa-2 xenograft model by protein capillary electrophoresis (Figure 3, Supplemental figure S1).

In MIA PaCa-2 xenograft tumors, CD147 protein expression levels remained unchanged at 24 h after h4^#^147D administration, but the level of protein expression at 72 h was decreased in a dose-dependent manner (Figure 3(a)).

Transmembrane proteins CD44, integrin *α*3, integrin *α*6, and MCT1 partially decreased at 24 and 72 h after administration of 1 or 3 mg/kg of h4^#^147D (Figures 3(b)–3(e)).

Focal adhesion kinase (FAK), an intracellular kinase that binds to CD44 and integrins, also decreased, and signals for the phosphorylated forms of FAK (Tyr397, Tyr925) and total FAK protein reduced in a similar manner at 24 and 72 h after h4^#^147D administration (Figures 3(f)–3(h)). Based on the observed inhibition of CD44/integrin *α*3/integrin *α*6/FAK in tumors with h4^#^147D treatment, h4^#^147D was considered to have caused cytoskeletal stress-mediated cell death in the tumor because these molecules are crucial for the organization of cytoskeletal proteins [40–42].

As one of the stress-responsive kinases, JNK has been reported to be phosphorylated and activated to induce cell death by repression of FAK [43], and the phosphorylation status of JNK was evaluated. The phosphorylation signal of JNK elevated at 24 and 72 h after administration of h4^#^147D at 1 or 3 mg/kg (Figure 3(i)). At 72 h, increases in phosphorylation of JNK were also observed at 72 h after h4^#^147D administration, and the effects appeared dose-dependent.

A substrate of JNK that is known to be increased and phosphorylated under JNK-activated conditions [44], c-Jun protein, was increased, and phosphorylation of c-Jun protein was also increased in groups treated with h4^#^147D at 1 and 3 mg/kg at 24 and 72 h after administration compared to each control group (Figure 3(j)), suggesting that JNK signal activation was induced in a dose-dependent manner in tumors after h4^#^147D administration.

Another stress-responsive kinase, p38MAPK, is reportedly activated by cellular stress along with JNK [45]. To examine the status of p38MAPK, we assessed heat shock protein 27 (HSP27), a known terminal substrate of the p38MAPK cascade that shows increased phosphorylation of HSP27 p38MAPK activation [46]. Phosphorylation of HSP27 was elevated at 24 and 72 h after h4^#^147D treatment with 1 and 3 mg/kg administrations (Figure 3(k)). We confirmed that p38MAPK activated cooperatively with JNK in tumors following h4^#^147D administration.

In addition to the JNK signal, activation of the SMAD signal including SMAD4 protein under stress by cytoskeletal protein disorganization has been reported [47]. According to a report by Denissova and Liu [48], since some functions of SMAD4 are independent of transforming growth factor (TGF)*β* and TGF*β* receptor, expressions of SMAD4 and one of the SMAD-downstream proteins, plasminogen activator inhibitor (PAI)-1 protein (SMAD-downstream protein that is induced at the mRNA transcriptional level by various factors, including SMAD signal) were assessed [49, 50]. As a result, the SMAD4 protein expression level was relatively higher in the MIA-PaCa-2 model than in other PDAC cancer cell line models (Supplemental figure S1(a)), and PAI-1 protein increased in the 3 mg/kg group at 72 h after h4^#^147D administration (Figure 3(l)).

Caspase-3 triggers apoptosis downstream of the activation of JNK, p38MAPK, and SMAD signals in a process of stress-mediated cell death, and caspase-3 is activated by protein processing with initiator caspases [51–53]. Levels of the active form of caspase-3, cleaved caspase-3, were significantly increased at 24 and 72 h after h4^#^147D administration (Figure 3(m)), suggesting that apoptosis was induced in h4^#^147D-treated tumors and that the observed tumor shrinkage in h4^#^147D-treated xenograft mice would therefore have been induced by apoptotic cell death with caspase-3 activation via activation of JNK, p38MAPK, and SMAD signals. In the MIA PaCa-2 xenograft model, the JNK inhibitor partially attenuated the antitumor effect (Supplemental figure S7), but the p38MAPK inhibitor did not in the same xenograft model treated with the previous candidate anti-CD147 antibody (Supplemental figure S8). SMAD4 expression levels correlated positively with antitumor effects of the previous candidate anti-CD147 antibodies (Supplemental figure S1). In addition, h4^#^147D showed a similar antitumor spectrum to previous candidate anti-CD147 antibodies in which limited antitumor effects were observed in SMAD4-negative PDAC models (Supplemental figure S1). Based on the efficacy data, JNK and SMAD4 might play more important roles in the antitumor effects of anti-CD147 antibodies than P38MAPK.

### 3.5. Inhibition of Antitumor Effects of h4^#^147D by KLF5

**Figure 4:**
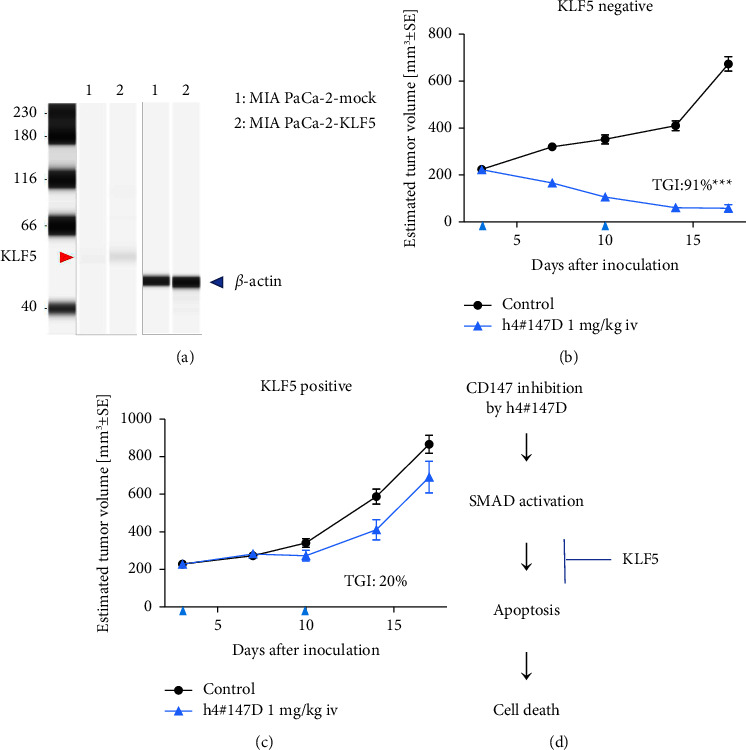
Inhibitory effects of KLF5 expression on h4^#^147D efficacy in the MIA PaCa-2 xenograft model. (a) Western blot image. KLF5 (red arrow) and *β*-actin (blue arrow) protein expression signals obtained from capillary protein electrophoresis for cell lysate of MIA PaCa-2 mock and MIA PaCa-2 KLF5 are shown. (b) Results of the mouse xenograft study for h4^#^147D. MIA PaCa-2 mock and MIA PaCa-2 KLF5 were inoculated subcutaneously into the immune-deficient mouse and used for evaluation of KLF5 function on h4^#^147D efficacy as KLF5-negative and KLF5-positive tumor models, respectively. (c) Asterisks indicate significant differences between the control group and antibody-treated groups (^*∗∗∗* ^*P* < 0.001). (d) Possible mechanism of KLF5-induced resistance to the.

KLF5 is known as a pleiotropic transcriptional factor involved in cell survival and induction of apoptosis via interactions with various other transcription factors, including SMAD4 [54, 55]. KLF5 thus represents a possible candidate for positive or negative involvement in the mechanism of action underlying the antitumor effects of anti-CD147 antibodies. With the aim of confirming the involvement of KLF5 in the antitumor activity of anti-CD147 antibodies, the in vivo antitumor activity of h4^#^147D was assessed using xenograft mice inoculated with either MIA-PaCa-2 KLF5 (MIA PaCa-2 cells expressing KLF5 generated by infection with a retrovirus inducing KLF5) or mock-infected cells, MIA PaCa-2 mock. Expression of KLF5 protein was confirmed for MIA-PaCa-2 KLF5 by western blot analysis, while no signal for KLF5 protein was detected from MIA PaCa-2 mock (Figure 4(a)). Results of the in vivo study are shown in Figures 4(b) and 4(c). The antitumor efficacy of h4^#^147D at 1 mg/kg was observed with 91% TGI in the MIA PaCa-2 mock xenograft model, while TGI by h4^#^147D in the MIA PaCa-2 KLF5 xenograft model was 20%. These results indicate that the antitumor effects of h4^#^147D are attenuated by expression of KLF5 in the MIA PaCa-2 xenograft tumor model, suggesting KLF5 as a potential negative regulator for the antitumor activity of h4^#^147D. The KLF5 expression profile in tumors may offer a possible predictive marker for h4^#^147D efficacy.

## 4. Discussion

In this study, we generated a humanized anti-CD147 antibody, h4^#^147D, and found that the h4^#^147D antibody showed strong antitumor effects with tumor regression in three SOC drug-insensitive mouse xenograft models using MIA PaCa-2 PDAC, Hep G2 HCC, and KU812 CML cell lines. h4^#^147D employed the IgG4 subtype with S228P mutation in the Fc-portion of the humanized antibody. IgG4 is known to possess reduced binding activity to antigens by the half-IgG exchange phenomenon with other IgG subtypes in the body, and exchange reactions are not thought to occur in the S228P mutant since the stability of the two forms is improved [32]. In addition, neither IgG4 exhibits antibody-dependent cytotoxic activity (unlike IgG1 antibodies), due to the weak binding to Fc*γ*RIIIa, nor does CDC with weak binding to C1q [56]. Indeed, h4^#^147D did not exhibit effector functions, ADCC, CDC, or ADCP activity against cancer cells (Table 3, Supplemental figures S3–S5), and the antitumor efficacy shown in murine xenograft models was suggested to be independent of immune-effector functions.

In xenograft tumor tissues after h4^#^147D treatment, reductions in levels of CD147 and its binding proteins (CD44, integrin *α*3, integrin *α*6, and MCT1) were observed. Reduction of FAK, an intracellular kinase, or its activated form, phosphorylated-FAK (which transduces the activated integrin signaling), was also seen following h4^#^147D treatment in this study. CD44 and those integrins bind to ECM, hyaluronic acid, and laminin, respectively, and modulate cytoskeletal control, cell migration, and survival in response to adhesion signals [57, 58]. JNKs and p38MAPK are stress-responsive kinases activated by various stressors or cytokine stimulations [45]. Activation of JNK has been reported to be induced by cytoskeletal stress induced by FAK inhibition, as well as by microtubule disorganization [28, 46, 47]. In addition, cytoskeletal stress reportedly induces SMAD signal activation [50]. The anti-CD147 antibodies obtained including h4^#^147D showed potent antitumor effects in the SMAD4-positive PDAC xenograft models but not in SMAD4-negative models (Supplemental Figure S1(s)). Moreover, an activated TGF*β*-SMAD downstream molecule, PAI-1, was also seen to be increased by h4^#^147D treatment, suggesting the possibility of dependence on SMAD4 status. Similar results in the Hep G2 anti-CD147antibody-sensitive cell line xenograft model suggested that increases in the same stress response signals and cleaved caspase-3 were induced by the h4^#^147D anti-CD147 antibody (Supplemental figure S6). In addition, the JNK inhibitor attenuated the antitumor effects of h4^#^147D (Supplemental figure S7) but not p38MAPK inhibitor (Supplemental figure S8). Such results may support the hypothesis that FAK inhibition induced by reducing CD147-binding molecules (such as CD44 and integrins) following anti-CD147 antibody treatment promotes cytoskeletal stress-dependent activation of JNK and SMAD4, leading to tumor cell death with caspase-3 activation.

KLF5 has been reported as an antiapoptotic factor that negatively regulates SMAD-dependent cell death [59, 60]. One of the h4^#^147D-sensitive cell line xenograft models, MIA PaCa-2, which was originally KLF5-negative, showed decreased sensitivity to h4^#^147D treatment following ectopic expression of KLF5 protein in this study. This suggests that KLF5 may work as a negative regulator for the antitumor effects of h4^#^147D. The BxPC-3 PDAC cell line xenograft model, which showed low sensitivity to anti-CD147 antibody treatment, was determined to be SMAD4-negative and KLF5-positive (Supplemental figures S1–S9). In addition, SMAD4-restoredBxPC-3 xenograft tumors showed higher sensitivity to anti-CD147 antibody treatment than SMAD4-negativeBxPC-3 xenograft tumors (Supplemental figure S10). These lines of evidence support the hypothesis that the KLF5 protein expression level and SMAD4 status could be possible biomarkers, which are predictive of tumor sensitivity to h4^#^147D. This may prove useful in selecting target cancer types and/or cancer patients in future clinical trials.

**Figure 5:**
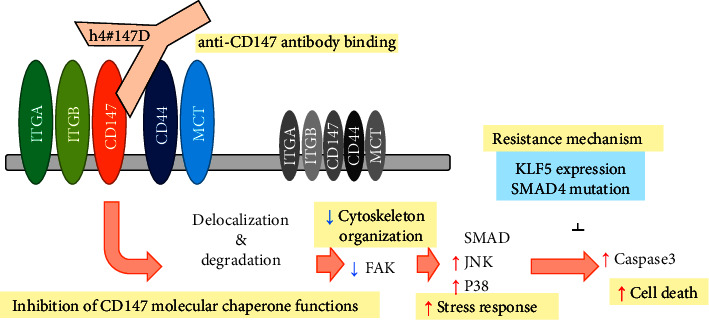
Graphical representation of a possible mechanism of action (MoA) for h4^#^147D anti-tumor efficacy. Administration of h4^#^147D leads to decreased cell surface expression of CD147 and its binding partners (such as integrins, CD44, and MCTs) via inhibition of CD147 molecular chaperone functions. Decreased or displaced CD44 and integrins may induce inhibition of FAK and cytoskeleton stress-mediated activation of stress-responsive signals such as SMAD, JNK, and P38MAPK. These activated stress-responsive signals lead to caspase-3 activation and cell death. Inhibitory mechanisms for the multiple stress-responsivesignal-induced cell death are possible mechanisms of resistance to the h4^#^147 mechanism of action.

So far, several anti-CD147 antibodies have been reported to exhibit antitumor activity in mouse models [61–63]. However, the antitumor activity of these antibodies is dependent on immune-effector function, and detailed analyses of the mechanisms of their antitumor activity have not been conducted. The mechanisms underlying tumor inhibition by CD147 antibodies revealed here or the reduced expression of CD147-binding proteins and the induction of stress-responsive cell death have not been described for any other anti-CD147 antibodies in previous reports. Moreover, no antibodies or small molecule inhibitors have been seen to simultaneously regulate CD44, integrins, FAK, and MCT1 in the same manner as h4^#^147D, suggesting that h4^#^147D exhibits strong antitumor activity with complete tumor regression via unique mechanisms of reducing CD147-binding proteins and activating stress-responsivesignal-mediated cell death. One possible mechanism of action for the antitumor effects of the anti-CD147 antibody is shown in Figure 5. For CD147-binding molecules that function to transmit signals intracellularly to extracellular ligands, localization at or near the plasma membrane is thought to be essential for proper functioning. These proteins become more severely dysfunctional with disruption by h4^#^147D than with inhibition by common inhibitors because proteins are no longer retained on the cell surface or near the plasma membrane, suggesting that h4^#^147D could exert strong antitumor effects that are not observed in other agents.

In particular, h4^#^147D showed high sensitivity to significantly intractable cancer types such as PDAC and HCC in some xenograft models. Since these cancers still have proven intractable with limited treatment options and remain difficult to cure even by the recent advances in drug development, our results warrant the further investigations whether this novel anti-CD147 antibody may have the potential to dissolve the current unmet needs for these intractable cancers.

## Figures and Tables

**Table 1:** Antibodies used for flow cytometric analysis.

Antibodies	Antibody supplier	Cat^#^
Anti-CD9-APC	Abnova	MAB4559
Anti-CD147-BV421	BD	562583
Anti-CD44-PE	Lifespan	LS-C46494
Anti-integrin*α*3/CD49c-PE	ThermoFisher Scientific	MA5-28112
Anti-integrin*α*6/CD49f-PE	Miltenyi Biotec	＃130-097-246

**Table 2:** Antibodies used for capillary electrophoresis-based western blot analysis.

Analysis protein	Antibody supplier	Cat^#^
*β*-Actin	Cell Signaling Technology	4967
CD147	Abcam	ab108317
CD44	R&D Systems	BBA10
Integrin *α*3	LSBio	LS-C660304-100
Integrin *α*6	R&D Systems	AF1350
MCT1	Santa Cruz Biotechnology	Sc-365501
Phospho-FAK (Tyr397)	Cell Signaling Technology	8556
Phospho-FAK (Tyr925)	Cell Signaling Technology	3284
FAK	Cell Signaling Technology	13009
Phospho-JNK (Thr183/Tyr185)	Cell Signaling Technology	9251
Phospho-c-jun (Ser73)	Cell Signaling Technology	3270
Phospho-HSP27 (Ser15)	Abcam	ab76313
Cleaved caspase-3	Cell Signaling Technology	9661

**Table 3:** Antibody profile of the anti-CD147 antibody, h4^#^147D.

Name of the antibody	h4^#^147D
Method of antibody generation for the parental nonhuman antibody	Myeloma fusion with rat lymphocytes derived from immunized rat with recombinant human CD147 protein
IgG subtype after humanization	Human IgG4 S228P (with deficiency in inter-H-chain bonds can be corrected by a single mutation: changing Ser228 (EU numbering) to pro [32])
Affinity to human CD147 (*K*_D_, SPR)	8.6 nM
Affinity to monkey CD147 (*K*_D_, SPR)	5.8 nM
Summary of epitope mapping using X-ray crystallographic structure	Hinge to the membrane-proximal immunoglobulin domain of CD147 isoform 2 (Arg106, Lys108 to Val110, Lys127 to Val134, and Gln164 to Gly165 in the amino acid sequence of human CD147 referred for Uniprot ID : P35613-2)

## Data Availability

All experimental raw data have been stored in Shinagawa R amp; D center of Daiichi Sankyo Co., Ltd, Tokyo, Japan.

## Abbreviations

SOC: Standard-of-care
ADCC: Antibody-dependent cellular cytotoxicity
CDC: Complement-dependent cell toxicity
ADCP: Antibody-dependent cell-mediated phagocytosis
Ig-LD: Immunoglobulin-like domain.

## Supplementary Materials

Supplementary Materials Figure S1: Summary of tumor inhibitory effects of anti-CD147 antibodies and CD147 and SMAD4 protein expression in PDAC cell line models. Figure S2: The CD147-Fab' complex in the asymmetric unit. Figure S3: Antibody-dependentcell-mediated cytotoxicity (ADCC) of anti-CD147 antibodies against Hep G2 cells. Figure S4: Complement-dependent cytotoxicity (CDC) of h4^#^147D using rabbit complement against Hep G2 cells. Figure S5: Antibody-dependent cellular phagocytosis (ADCP). Figure S6: Western blot analyses of tumor lysates from a HCC xenograft tumor model with anti-CD147 antibody treatment. Figure S7: The inhibitory effect of a JNK inhibitor for h4^#^147D efficacy in the MIA PaCa-2 xenograft model. Figure S8: The inhibitory effect of a p38MAPK inhibitor for efficacy of an anti-CD147 antibody, LN22R8IgG4P (human IgG4 Fc chimeric murine monoclonal antibody), in the MIA PaCa-2 xenograft model. Figure S9: KLF5 expression in PDAC cell lines. Formalin-fixedparaffin-embedded PDAC cell lines, MIA PaCa-2 and BxPC-3, were analyzed immunohistochemically with an anti-KLF5 antibody. Figure S10: SMAD4 function in antitumor effects of the anti-CD147 antibody..
