# Supplementary material for: Novel Antibody Exerts Antitumor Effect through Downregulation of CD147 and Activation of Multiple Stress Signals
Source: J Oncol. 2022 Nov 4;2022:3552793. doi: 10.1155/2022/3552793 (PMC9652086; doi:10.1155/2022/3552793)
Supplement: Supplementary Materials — Figure S1: Summary of tumor inhibitory effects of anti-CD147 antibodies and CD147 and SMAD4 protein expression in PDAC cell line models. Figure S2: The CD147-Fab' complex in the asymmetric unit. Figure S3: Antibody-dependentcell-mediated cytotoxicity (ADCC) of anti-CD147 antibodies against Hep G2 cells. Figure S4: Complement-dependent cytotoxicity (CDC) of h4#147D using rabbit complement against Hep G2 cells. Figure S5: Antibody-dependent cellular phagocytosis (ADCP). Figure S6: Western blot analyses of tumor lysates from a HCC xenograft tumor model with anti-CD147 antibody treatment. Figure S7: The inhibitory effect of a JNK inhibitor for h4#147D efficacy in the MIA PaCa-2 xenograft model. Figure S8: The inhibitory effect of a p38MAPK inhibitor for efficacy of an anti-CD147 antibody, LN22R8IgG4P (human IgG4 Fc chimeric murine monoclonal antibody), in the MIA PaCa-2 xenograft model. Figure S9: KLF5 expression in PDAC cell lines. Formalin-fixedparaffin-embedded PDAC cell lines, MIA PaCa-2 and BxPC-3, were analyzed immunohistochemically with an anti-KLF5 antibody. Figure S10: SMAD4 function in antitumor effects of the anti-CD147 antibody.. [file 3552793.f1.docx]

**Supporting information**

**Antibody generation and screening**

About 20,000 monoclonal hybridoma were generated using splenocytes and iliac lymph node from 4 to 6 weeks old murine (BALB/cAnNCrlCrlj, Charles river, Japan) and 7 weeks old rats (WKY/Izm, SLC, Shizuoka, Japan) immunized with up to 5 times of 5×10^6^ to 1×10^7^ cells of LNCaP (prostate cancer), MCF7(breast cancer), AsPC-1 (pancreatic cancer) and PANC-1 (pancreatic cancer) human cancer cell lines using ClonaCell-HY Hybridoma Kit (STEMCELL Technologies, Vancouver, Canada) with murine myeloma cell line, P3X63Ag8U or SP2/0-Ag14 (ATCC, Virginia, US). Aiming to protect the proteins that are the antigen, in the preparation of the cells for immunization, Versene solution (Thermo Fisher scientific, Massachusetts, US) was used as a gentle non-enzymatic cell dissociation reagent at the cell dissociation process from culture flasks. Eight anti-CD147 antibodies were screened from those hybridoma supernatants by *in vitro* cell killing assay and direct ELISA using immobilized human CD147-Fc protein. The 2P10F2 and LN22R8 anti-CD147 antibodies showed potent anti-tumor effects in CD147- and SMAD4 protein-positive pancreatic cancer xenograft models (Supplemental figure S1), however those two anti-CD147 antibodies did not display cross-reactivity with rodent or monkey CD147, preventing the necessary safety testing. As a result, about 3000 monoclonal hybridomas were additionally generated from rats immunized with human recombinant CD147 protein by electrofusion using LF301 Cell Fusion Unit (BEX, Tokyo, Japan) with murine myeloma cell line, SP2/0-Ag14. A recombinant human CD147 protein (Creative BioMart, Shirley, NY, US) retaining a three-dimensional structure which could interfere the binding of pre-obtained anti-CD147 antibodies to human cancer cell expressing CD147 in flowcytometry analysis was used as immunogen to generate hybridoma producing anti-CD147 antibodies. Forty anti-CD147 rat monoclonal antibodies showing human and monkey CD147 cross-reactivity were screened by flow cytometry using human- and monkey-CD147-expressing CHO-K1 cells. Based on the anti-tumor efficacy of the 40 anti-CD147 antibody in the MIA-PaCa-2 pancreatic cancer cell line xenograft model, 5 anti-CD147 antibodies displaying cross-reactivity with monkey-CD147 were selected for humanization of the antibody sequence.

**A**

**B**


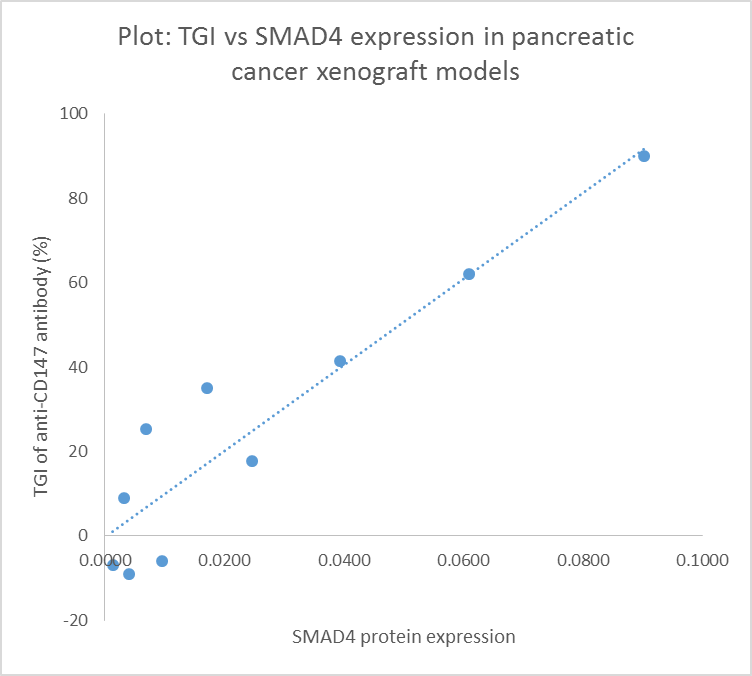

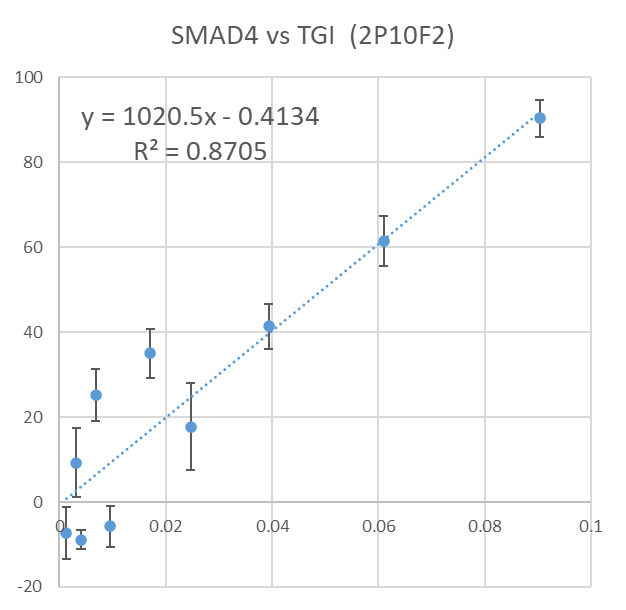


Normalized SMAD4 protein expression

P = 0.0012

Figure S1. Summary of tumor inhibitory effects of anti-CD147 antibodies and CD147 and SMAD4 protein expression in pancreatic cancer cell line models. A) Anti-tumor effect of anti-CD147 antibodies 2P10F2 (rat IgG2b) and LN22R8 (mouse IgG3) were determined in 10 pancreatic cancer cell line xenograft models. Anti-tumor effect was evaluated in nude- or NOD SCID mice bearing each pancreatic cancer cell line tumor (n=5 or 6) with intravenous administration at 10 mg/kg in comparison to non-treated mice bearing the same pancreatic cancer cell line tumor (n=5 or 6). The percentage tumor growth inhibition (TGI) by anti-CD147 antibodies was calculated in the same way as other xenograft studies in the main figures, by comparison with tumor size in control mice without antibody treatment. Protein expression levels of CD147 and SMAD4 were determined by Western blot analysis for pancreatic cancer cell line lysates from in vitro culture using the same simple Western system used in Figure 3 of the main text. B) Plot of anti-tumor efficacy of the anti-CD147 antibody 2P10F2 and SMAD4 protein expression level in 10 pancreatic cancer cell lines. The two anti-CD147 antibodies, LN22R8 and 2P10F2, are previous candidates that only bind to human CD147 protein, not to rodent or monkey CD147 protein. These antibodies therefore have not been advanced to the humanization process, as evaluation of the safety profile in monkeys can not be tested. Correlation analysis was conducted between anti-tumor efficacy of 2P10F2 antibody and expression level of SMAD4 protein in the pancreatic cancer cell lines.

Two complex structures in the asymmetric unit

Figure S2. CD147-Fab' complex in the asymmetric unit. Two copies of each of the CD147 (magenta), heavy chain (cyan), and light chain (green) are present. The IgC2 domain of CD147 could not be modelled in one of the complexes, due to unresolved electron density attributed to the flexibility afforded by the hinge region.


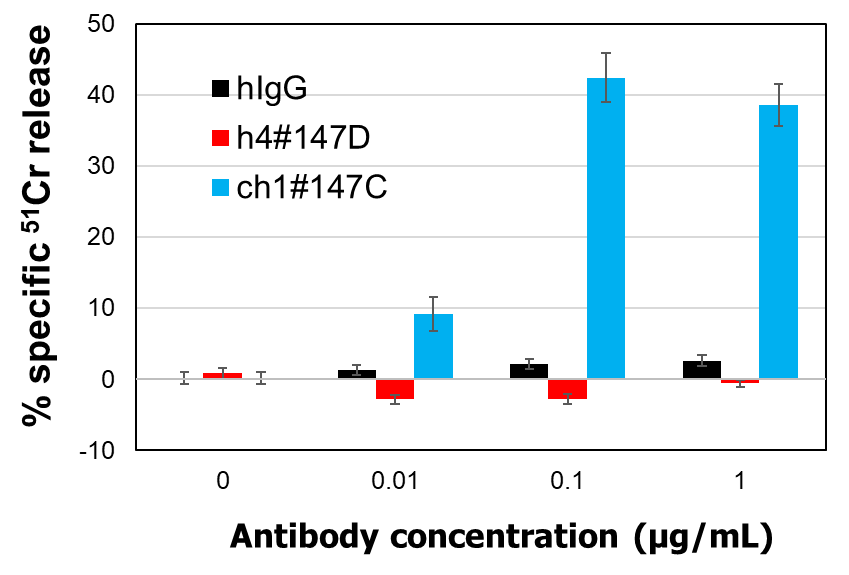


Figure S3. Antibody-dependent cell-mediated cytotoxicity (ADCC) of anti-CD147 antibodies against Hep G2 cells. The ADCCs of the anti-CD147 antibodies h4#147D and ch1#147C (another anti-CD147 antibody with human IgG1 Fc) using human PBMCs against Hep G2 cells were are evaluated by a 4-h ^51^Cr release assay in the presence of 0.01–1 μg/mL of antibody with an E/T ratio of 20.


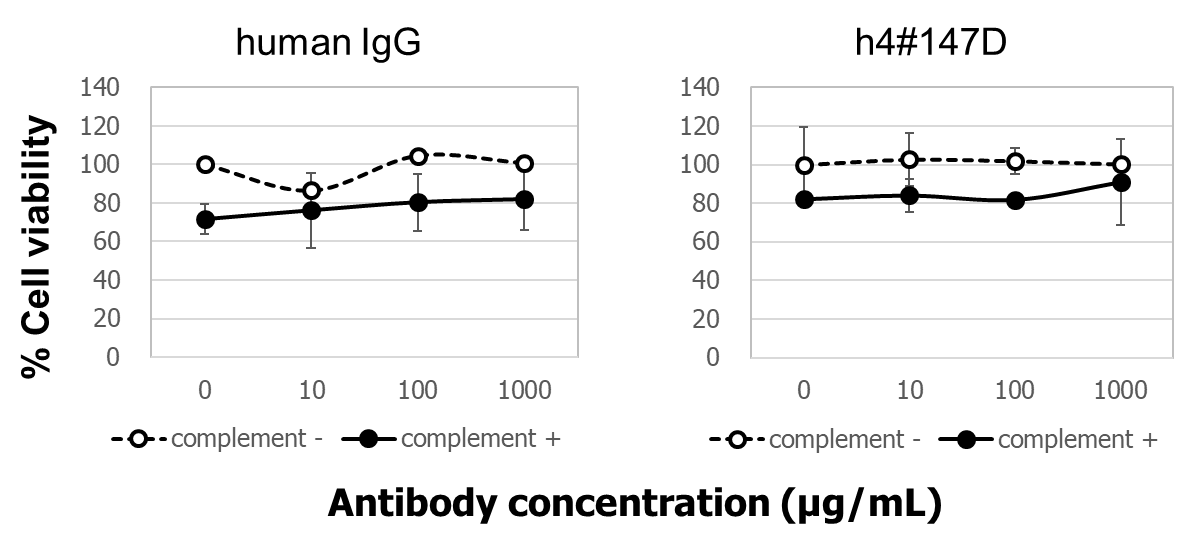


Figure S4. Complement-dependent cytotoxicity (CDC) of h4#147D using rabbit complement against Hep G2 cells. CDC was evaluated by cell viability assay using the CellTiter-Glo® Luminescent assay (Promega) in the presence of 0.01–10 μg/mL of antibody. No CDC activity of h4#147D was detected.


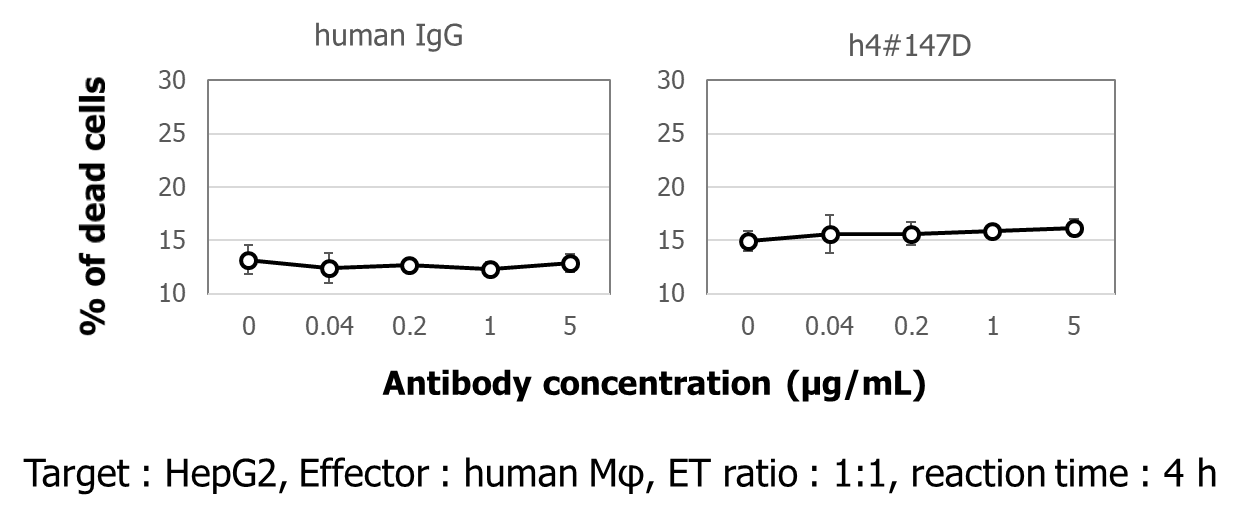


Figure S5. Antibody-dependent cellular phagocytosis (ADCP). ADCP flow cytometry analysis of human macrophage cells co-cultured with Hep G2 cells at a 1:1 ratio with human IgG or h4#147D in the range of 0.04–5 μg/ml. The graph depicts the percentage of ADCP-positive macrophages from three independent experiments. No ADCP activity of h4#147D was detected.


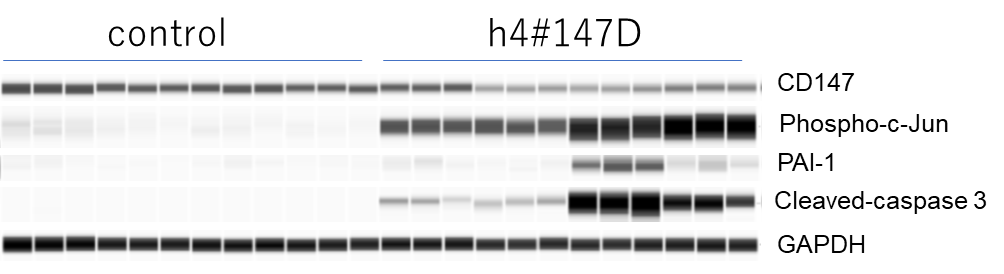


Figure S6. Western blot analyses of tumor lysates from a liver cancer xenograft tumor model with anti-CD147 antibody treatment. Tumor lysates prepared from Hep G2 liver cancer cell line xenograft tumors (n=12 per group) with administration of h4#147D antibody at 10 mg/kg-iv in 48 h were analyzed by Western blot analysis using anti-CD147 antibody, anti-phospho-c-Jun antibody, anti-PAI-1 antibody, anti-cleaved-caspase 3 antibody and anti-GAPDH antibody. Tumor lysates from vehicle-treated Hep G2 tumors were used as controls.


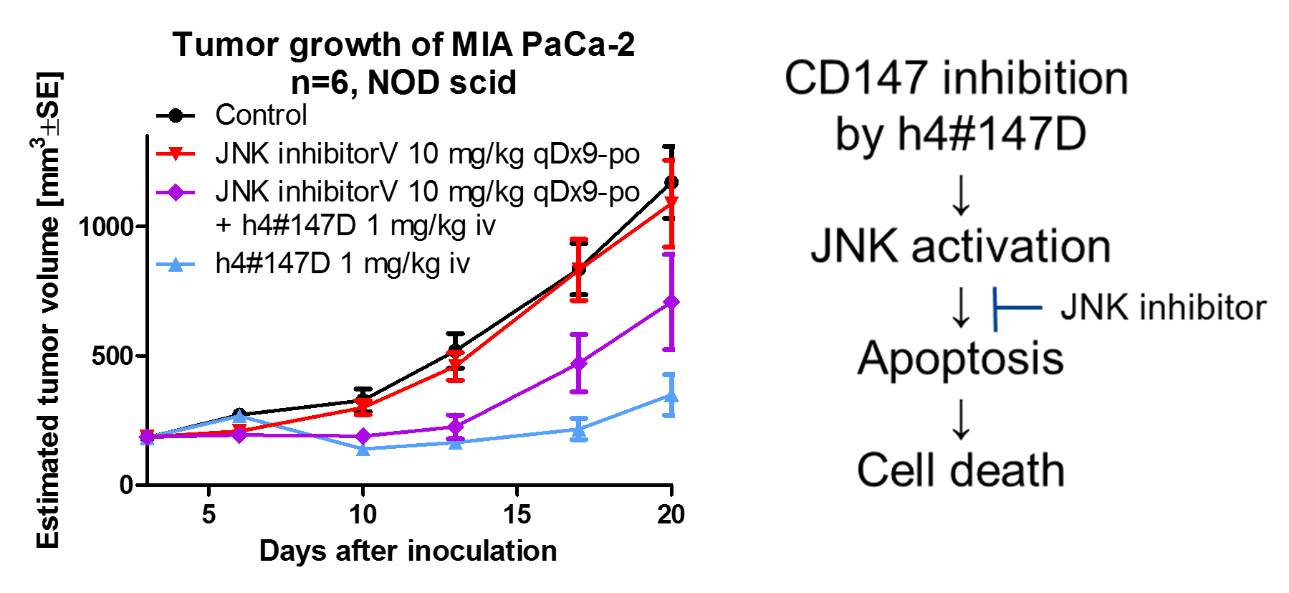


Figure S7. Inhibitory effects of a JNK inhibitor for h4#147D efficacy in the MIA PaCa-2 xenograft model. Five-week-old NOD SCID mice were subcutaneously injected with MIA PaCa-2. After the tumor volume reached 200 mm^3^, animals were randomized into groups of 6 mice and treated with either oral JNK inhibitor V at 10 mg/kg/day for 9 days, 1 mg/kg body weight of h4#147D (single intravenous injection) or a combination of JNK inhibitor and h4#147D. Non-treated animals were used as controls. Data show mean and SEM.

Figure S8. Inhibitory effects of a p38MAPK inhibitor on efficacy of an anti-CD147 antibody, LN22R8IgG4P (human IgG4 Fc chimeric murine monoclonal antibody), in the MIA PaCa-2 xenograft model. Five-week-old NOD SCID mice were subcutaneously injected with MIA PaCa-2. After the tumor volume reached approximately 200 mm^3^, animals were randomized into groups of 5 mice and treated with either oral p38MAPK inhibitor (PH797804) at 10 mg/kg/day for 8 days, 1 mg/kg body weight LN22R8IgG4P (intraperitoneal, qWx2 on days 7 and 14), or a combination of p38MAPK inhibitor and LN22R8IgG4P. Non-treated animals were used as controls. Data show mean and SEM.


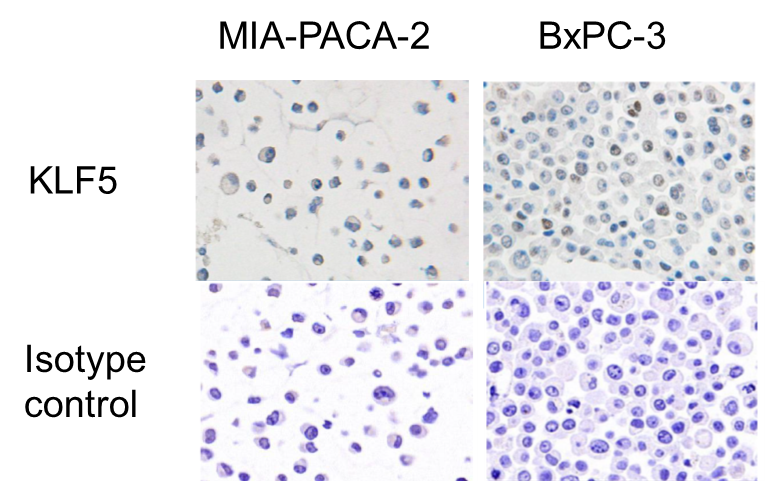


Figure S9. KLF5 expression in pancreatic cancer cell lines. Formalin-fixed paraffin-embedded pancreatic cancer cell lines, MIA-PaCa-2 and BxPC-3, were analyzed immunohistochemically with anti-KLF5 antibody.


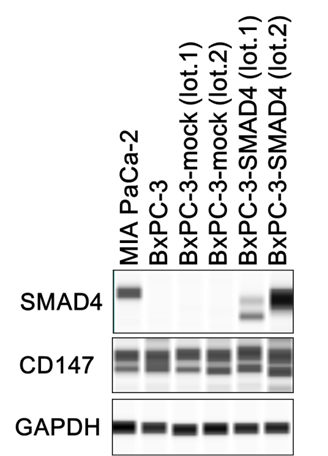
**A**

**B**


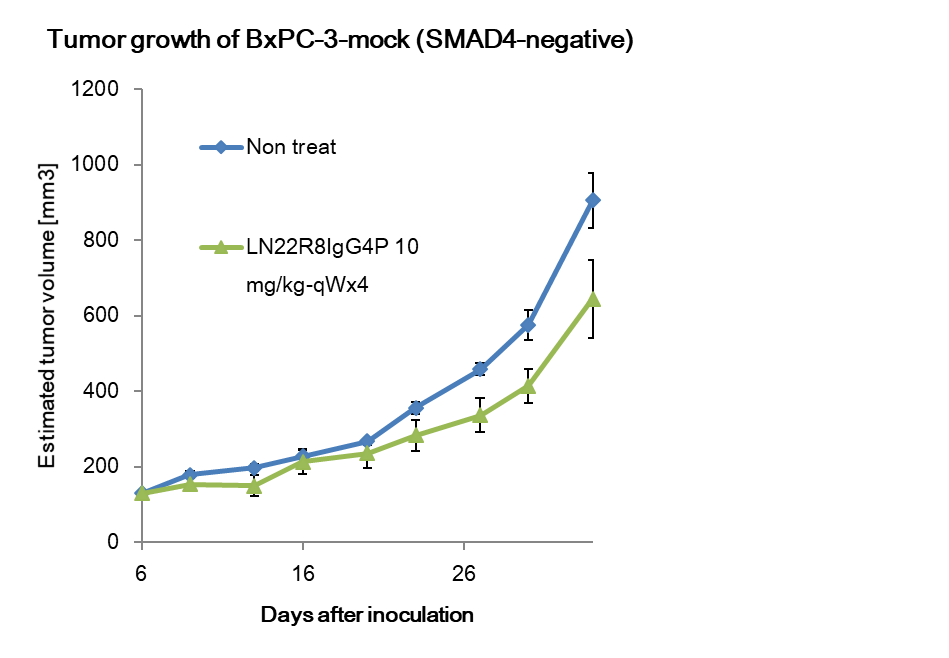


**C**

**
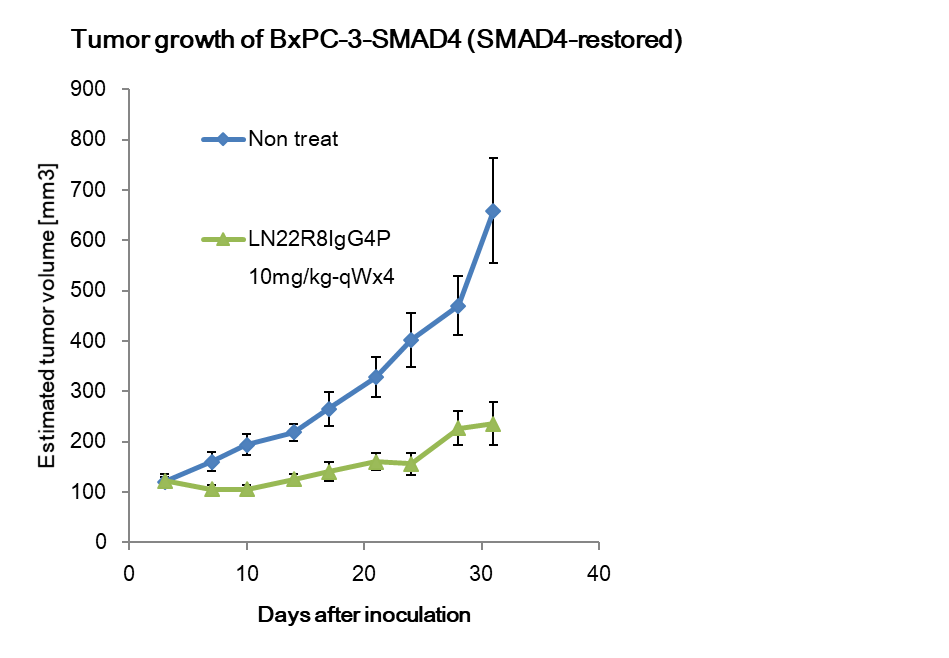
**

Figure S10. Effects of SMAD4 function on anti-tumor effects of anti-CD147 antibody. A) Western blot analysis for protein lysates from the MIA-PaCa-2 and BxPC-3 pancreatic cancer cell lines. Both cell lines are CD147-positive. SMAD4 status was positive in MIA PaCa-2 and negative in BxPC-3. BxPC-3 cells with retroviral infection using an SMAD4-expressing retrovirus vector showed restored expression of SMAD4 protein in cells. BxPC-3-mock (lot 2) and BxPC-3-SMAD4 (lot 2) were used as SMAD4-negative and -positive pancreatic cancer cell line models, respectively, for the subsequent xenograft studies. As anti-CD147 antibody, LN22R8IgG4P (human IgG4 Fc chimeric murine monoclonal antibody) was used for the following xenograft studies. B) Tumor growth of the BxPC-3 mock xenograft model in nude mice (n=5) with or without anti-CD147 antibody treatment at 10 mg/kg-iv. qWx4. C) Tumor growth of the BxPC-3-SMAD4 xenograft model in nude mice (n=5) with or without anti-CD147 antibody treatment at 10 mg/kg-iv. qWx4.
